# Biochemical and immunological investigation of fascioliasis in cattle in Egypt

**DOI:** 10.14202/vetworld.2020.923-930

**Published:** 2020-05-18

**Authors:** Nani Nasreldin, Rania Samir Zaki

**Affiliations:** 1 Department of Pathology and Clinical Pathology, Faculty of Veterinary Medicine, New Valley University, El-Kharga, P.O. Box 72511, Egypt; 2 Department of Food Hygiene, Faculty of Veterinary Medicine, New Valley University, El-Kharga, Egypt

**Keywords:** cattle, *Fasciola hepatica*, fascioliasis, immune and biochemical response, liver damage

## Abstract

**Background and Aim:**

*Fasciola hepatica* and *Fasciola gigantica* are two commonly reported liver flukes that cause fascioliasis in ruminants. Among the members of the genus *Fasciola*, *F. hepatica* was identified in the study area. Fascioliasis is a major disease that affects the production of livestock by causing liver damage. *F. hepatica* has developed advanced mechanisms to trick, elude, and alter the host immune response, similar to an extrinsic stressor. These mechanisms consequently affect the animals’ physiological and metabolic functions *in vivo* and postmortem changes, which have significant influences on animal welfare and meat quality development. Therefore, this study aimed to determine the current prevalence of cattle fascioliasis at abattoirs in El-Kharga city, New Valley Governorate, Egypt, and to investigate the changes in serum biochemical and immunological parameters and oxidative stress factors due to *Fasciola* spp. infection in terms of meat quality and immune response.

**Materials and Methods:**

A total of 226 cattle were inspected for the presence of *Fasciola* spp. The liver of each cattle was examined by making several incisions for detecting adult *Fasciola* spp. in El- Kharga. The blood samples were collected to analyze the changes in serum biochemical and immunological parameters and oxidative stress factors.

**Results:**

Of the 226 cattle, 38 (16.81%) were positive for *F*. *hepatica* at the postmortem examination. Cattle infected with *F*. *hepatica* had highly elevated serum alanine aminotransferase, aspartate aminotransferase, glutamate dehydrogenase, γ-glutamyl transferase, urea, and creatinine levels. Immunological cytokine profiles showed significantly increased serum interleukin (IL)-4, IL-10, and transforming growth factor-beta levels and a significantly decreased interferon-γ level. Furthermore, oxidative stress profiles showed significantly increased serum malondialdehyde and nitric oxide levels and significantly decreased total antioxidant capacity and reduced glutathione level.

**Conclusion:**

This study demonstrated that *F. hepatica* infection alone is an oxidative stress factor that affects slaughtered animals, leading to biochemical and metabolic alterations in the early postmortem period.

## Introduction

Fascioliasis is a parasitic disease caused by the liver flukes *Fasciola hepatica* and *Fasciola gigantica*, which are digenetic trematodes identified in most regions worldwide [1,2]. Fascioliasis is also an enzootic disease occurring in farm animals in Egypt, where it is considered an emerging public health problem. In addition, the General Organization of Veterinary Services in Egypt has estimated that *Fasciola* spp. accounts for 30% of loss in milk and meat production [3]. These parasites infect the liver of cattle, due to which the animals are orderly condemned (i.e., officially declared unfit) on inspection at slaughterhouses. In fact, the liver is the most specific and important offal condemned due to fascioliasis due to the preventable lesions or diseases that are distinguished pending during routine postmortem meat inspections [4]. The analysis of individual cattle and meat quality is a compulsory process that must be performed on the liver condemnation at abattoirs and is ordinarily used by farmers and veterinarians as an indicator of the severity of fascioliasis on a farm [5]. The lesions in the liver are caused due to the toxic and mechanical effects of liver flukes, the excretory products of the parasites, and the decomposed products of *Fasciola* spp. in the bile and liver tissues, all of which affect the complex vascular and biliary system in the liver [6].

Hepatic enzymes such as alanine aminotransferase (ALT), aspartate aminotransferase (AST), glutamate dehydrogenase (GDH), and γ-glutamyl transferase (GGT), which are used for diagnosing hepatobiliary diseases, are highly concentrated in the liver. Any alteration in the integrity of the cell membrane of hepatocytes or the presence of necrosis in the liver cells or biliary epithelium would result in leakage of these enzymes into the serum, which subsequently elevates their serum levels [7]. *Fasciola* spp. infection causes the release of reactive oxygen species (ROS) that damage the cell wall and cause hepatic tissue necrosis, with subsequent release of enzymes into the blood, thereby increasing their concentration above the physiological level [8]. In *Fasciola* spp. infection, the bile accumulated in bile ducts due to biliary cholestasis is one of the causes of oxidative stress, wherein biliary cholestasis constitutes a source for the production of hydroperoxides (OH˙), superoxide anion (O_2_^-^), and hydrogen peroxide (H_2_O_2_). Furthermore, liver fibrosis is another cause of oxidative stress through enhanced production of free radicals. Inflammation of the hepatobiliary system due to fascioliasis is also associated with eosinophils, neutrophils, and macrophages, which are potent sources of oxidants that not only target parasites but also cause tissue damage [9]. Liver flukes are considered efficient immunomodulators that produce several effector molecules that can aid in exploiting the host immune response to ensure their development and survival as well as maintenance of infection, which ultimately results in cellular and tissue damage [10,11]. In addition, fascioliasis is a powerful inducer of Th2 responses. This process is accomplished within 24 h following oral infection, wherein peritoneal macrophages express Th2-associated markers and display a reduced ability to respond to Th1 stimulants, with subsequent impairment of the host ability to manifest any effective Th1 responses against bacteria and other pathogens. This rapid and potent immunosuppression imposed by *Fasciola* spp. explains why infected hosts do not develop resistance against these parasites [12].

Against this background, the present study aimed to determine the current prevalence of bovine fascioliasis at abattoirs in El-Kharga city, New Valley Governorate, Egypt, to determine the changes in serum biochemical and immunological markers and oxidative stress factors imposed due to *Fasciola* spp. infection in terms of meat quality and immune response, and to delineate the effect of fascioliasis on immune response by investigating the serum cytokine profile (interleukin [IL]-4, IL-10, transforming growth factor-beta [TGFβ]1, and interferon-γ [IFN-γ]).

## Materials and Methods

### Ethical approval

This study was performed according to the regulation and procedures approved by the Ethics Committee on Animal Experimentation of the Faculty of Veterinary Medicine, New Valley University, and the guide for the care and use of animals (National Institute of Health Publication No. 8023, revised 1978).

### Study period and study area

This study was conducted from January 2018 to December 2018 in El-Kharga city, New Valley Governorate, Egypt, located on the west of Nile Valley between 25°26′56″ N latitude and 30°32′24″ E longitude. El-Kharga Oasis is located southwest of Egypt and is situated 600 km to the south of Cairo Governorate.

### Experimental design

The El-Kharga Municipal abattoir was visited throughout the study period. A total of 226 cattle were inspected during postmortem examination, with collection of the blood samples before slaughtering.

### Sample collection

The blood samples were collected in plain vacutainers and placed at an inclined position for 20 min at room temperature. Thereafter, they were placed in the refrigerator to avoid glycolysis and complete clot retraction. Then, the samples were centrifuged at 3000 rpm for 10 min until the clear serum was separated, which was carefully collected and stored in Eppendorf tubes at −80°C until biochemical assessments.

#### Slaughterhouse study

An active slaughterhouse survey was conducted using a cross-sectional study design during the postmortem examination of randomly selected cattle slaughtered at the El-Kharga abattoir.

During the antemortem examination, the age and sex of the animal were recorded according to the Egyptian legislations for meat inspection, wherein slaughtering of cattle and female bovines is not allowed before all the permanent teeth have erupted(over a period of 5 years), whereas male bovines and cattle are approved for slaughtering after 2 years.

During the postmortem examination, each liver was superficially inspected, palpated, and incised according to the routine meat inspection procedure described by Yatswako and Alhaji [13].

Based on antemortem and postmortem examinations, ten apparently healthy cattle and not suffering from any health problems with apparently normal livers were kept as normal healthy control.

All the livers harboring *Fasciola* spp. that were condemned, were recorded, and the flukes were collected for species identification, as described by Martínez-Pérez [14].

#### Serum biochemical analysis

ALT, AST, urea, and creatinine levels were analyzed using commercial kits (Human Co. Germany). Oxidative stress was determined by measuring the serum glutathione (GSH), malondialdehyde (MDA), and nitric oxide (NO) levels and the total antioxidant capacity (TAC) using commercial kits (Biodiagnostic Egypt). All serum biochemical and oxidative stress parameters were measured spectrophotometrically (5010 V5+, photometer, RIELE Co. Germany), according to the manufacturer’s instructions.

GDH and GGT levels were determined using an enzyme-linked immunosorbent assay kit for GDH level was determined, Organism Species: Bovine (Cattle), Cloud-Clone Corp, USA. Enzymatic kinetic assay for quantitative determination of serum GGT (Pointe Scientific, USA) concentration was conducted spectrophotometrically (5010 V5+, photometer, RIELE Co. Germany), according to the manufacturer’s instructions.

#### Immunological studies

Immunological parameters (IFN-γ, IL-4, IL-10, and TGFβ1) were assayed using bovine IFN-gamma and bovine IL-4 ELISA Kit (RayBiotech, USA) and bovine IL-10 and bovine TGFβ1 ELISA Kits (Cusabio, USA).

### Statistical analysis

Data on the serum levels of biochemical, immunological, and oxidative stress parameters were statistically analyzed by independent samples t-test using SPSS Version 23.0 (IBM Corp. Armonk, NY, USA).

## Results

**Figure-1:**
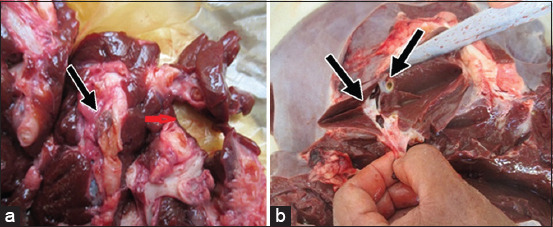
The macroscopic examination detected the liver fluke in the liver of cattle carcasses. (a and b) the number of the adult parasites and the presence of cirrhosis, cholangitis, cholangitis purulenta, cirrhosis hepatitis in the postmortem inspection.

Of the total 226 liver samples obtained from the cattle slaughtered at the El-Kharga abattoir, 38 (16.81%) were positive for *F. hepatica*, as revealed by the postmortem examination (Figure-1). The two highest percentages of infections were recorded in May 2018 (26.67%) and December 2018 (23.08%).

Serum biochemical parameters profile in *Fasciola*-infected cattle samples (infected group) if compared to control group evidenced a significant increase in serum ALT (30.85±1.24 vs 16.98±0.49, p<0.001), AST (100.44±1.04 vs 76.97±0.87, p<0.001), GDH (141.89±3.87 vs 45.38±0.67, p<0.001), GGT (57.22±1.81 vs 20.65±0.76, p<0.001), urea (34.70±1.35 vs 10.20±0.93, p<0.001) and creatinine (1.44±0.05 vs 0.95±0.02, p<0.05) (Table 1).

**Table-1:** Some serum biochemical profiles (Mean±S.E), for normal (Control group) and *Fasciola hepatica* infected cattle (Infected group).

Biochemical parameters	Control group	Infected group
ALT (U/L)	16.98±0.49	30.85±1.24**
AST (U/L)	76.97±0.87	100.44±1.04**
GDH (ng/ml)	45.38±0.67	141.89±3.87**
GGT (U/L)	20.65±0.76	57.22±1.81**
Urea (mg/dl)	10.20±0.93	34.70±1.35**
Creatinine (mg/dl)	0.95±0.02	1.44±0.05*

Values represent mean±SE. SPSS version 23 (Independent sample t-test). Significance:

* p<0.05,

** p<0.001 comparing to control cattle. γ-GT=γ-glutamyltransferase, GD=Glutamate dehydrogenase, AST=Aspartate aminotransferase, ALT=Alanine aminotransferase, SE=Standard error

The immunological cytokine profile revealed that the serum IL-4 (325.27±10.73 vs 44.87±1.12, p<0.001), IL-10 (27.79± 0.80 vs 7.85±0.09, p<0.001) and TGFβ1 (41.29± 0.59 vs 11.41±0.55, p<0.001) levels were significantly increased in infected group in comparison with control group. However, serum IFN-γ (0.58± 0.01 vs 0.82±0.01, p<0.001) level was significantly decreased in the infected group as compared with control group (Table-2).

**Table-2:** Cytokine profiles (Mean±S.E), for normal (Control group) and *Fasciola hepatica* infected cattle (Infected group).

Cytokine profile	Control group	Infected group
IL-4 (pg/ml)	44.87±1.12	325.27±10.73*
IL-10 (pg/ml)	7.85±0.09	27.79± 0.80*
IFN-γ (ng/ml)	0.82±0.01	0.58± 0.01*
TGF-β1 (ng/ml)	11.41±0.55	41.29± 0.59*

Values represent mean±SE. SPSS version 23 (Independent sample t-test). Significance:

* p<0.001 comparing to control cattle. IL=Interleukin, IFN-γ=Interferon-γ, TGF-β1=Transforming growth factor-beta, SE=Standard error

The analysis of the oxidative stress profile of the infected cattle revealed a significant increase in the serum MDA (4.29±0.19 vs 2.99±0.05, p<0.01) and NO (137.78±1.27 vs 71.73±1.78, p<0.001) levels, whereas the serum GSH (0.46±0.12 vs 2.30±0.04, p<0.001) and TAC (0.78±0.02 vs 1.77±0.43, p<0.001) concentrations were significantly reduced in comparison with control group (Table-3).

**Table-3:** Oxidative stress profiles for normal (Control group) and *Fasciola hepatica* infected cattle (Infected group).

Oxidative stress parameter	Control group	Infected group
MDA (nmol/ml)	2.99±0.05	4.29±0.19*
NO (µmol/L)	71.73±1.78	137.78±1.27**
GSH (mg/dL)	2.30±0.04	0.46±0.12**
TAO (mM/L)	1.77±0.43	0.78±0.02**

Values represent mean±SE. SPSS version 23 (Independent sample t-test). Significance:

* p<0.01,

** p<0.001 comparing to control cattle. GSH=Glutathione, NO=Nitric oxide, MDA=Malondialdehyde, SE=Standard error

## Discussion

Fascioliasis is an extremely serious disease that causes enormous losses in cattle production and in the related meat, milk, and leather industry as *Fascciola* spp. could be found in ectopic location as the skin[15]. Although the prevalence of fascioliasis recorded in the present study was low (16.81%), which may not be a regular consideration of its prevalence in New Valley, Egypt, analysis of the different immunological and biochemical parameters emphasizes on how the infestation of *F. hepatica* even at such a low prevalence rate affects the quality of meat and meat products, particularly when apparently clinically normal animals are allowed to be slaughtered after antemortem examination.

Abattoirs are important in terms of assurance of the quality of meat and meat products for human consumption. Furthermore, they provide byproducts for livestock-based industries. More importantly, abattoirs aim at controlling animal and zoonotic diseases. The investigation of bovine fascioliasis at abattoirs has provided useful information regarding the prevalence of diseases and the economic losses caused due to liver condemnation worldwide [13].

In the present study, the overall prevalence rate of bovine fascioliasis was 16.81% (38/226 samples) in the cattle slaughtered at the El-Kharga abattoir. Nearly, equivalent results have been reported by Ejeh *et al*. [16] 14.56% (9478/64,978 samples) at Makurdi, Nigeria, Okaiyeto *et al*. [17] 14.05% (73,761 samples), and Raji *et al*. [18] 14.16% (1,022/7,217 samples at Zaria, Nigeria. However, Odigie and Odigie [19] reported a low prevalence rate of bovine fascioliasis at 3.33% (180/540 samples) at Egor, Nigeria.

In the present study, the highest prevalence rate (26.67%, 4/15 samples) of *Fasciola* spp. infection was observed in May 2018, which may be due to the types of dry feeds provided during summer, wherein the animals are fed hay or straw rice after completion of providing green feed in winter and spring. This finding is consistent with that reported by Boray [20], who concluded that inadequately dried hay stored under cold and wet conditions could harbor the metacercaria of *F. hepatica*. In addition, Kato *et al*. [21] indicated a markedly higher prevalence rate in Japanese native cattle than in Friesian cattle, possibly due to the difference in cattle feed, given that Japanese native cattle were fed rice straw; they stated that rice straw feed could be related to the high prevalence rates of cattle fascioliasis in Japanese native cattle. However, the high prevalence rate at 23.08% (3/13 samples) observed in December 2018 in the present study may be due to grazing on green pastures (especially containing berseem), where the metacercaria could have encysted despite El-Kharga city being a dry region and having a higher temperature (24±2°C) than the neighboring regions. Mas-Coma *et al*. [22] reported that the first transmission of *Fasciola* spp. in Egypt was by a snail that was not related to the family Lymnaeidae (*Biomphalaria alexandrina*, and *Planorbidae*); this snail needs swampy areas such as low streaming lakes that allow sufficient moisture for the survival of infective metacercariae [23].

In the present study, only *F. hepatica* was detected at the study site (100%), as revealed by postmortem examination, whereas *F. gigantica* and mixed specifies were not detected, which is consistent with that reported by Tulu [24], who stated that *F. hepatica* (100%) was the predominant species of zoonotic liver flukes found at an abattoir in Ambo district, Ethiopia. The prevalence rate of *F. hepatica* can reach up to 77% in developing countries [15].

In the present study, significant increases were observed in the serum ALT, AST, and GDH levels in the cattle infected with *F*. *hepatica*, possibly due to the lesions in the liver. Our results are consistent with those reported by Kowalczyk *et al*. [1] and Jarujareet *et al*. [25] who described that the significantly elevated serum ALT, AST, and GDH levels are related to the inflammatory state of the liver in addition to the continuous mechanical and enzymatic injury as well as the tissue destruction caused by migration of liver flukes through the hepatic parenchyma toward the bile duct. Furthermore, *Fasciola* spp. induce the release of ROS, causing subsequent damage to the cell wall and leading to hepatic tissue necrosis [26].

The highly elevated serum GGT level observed in our study is in agreement with that reported by Yang *et al*. [27], who explained that the initial increase in plasma GGT level is due to the penetration of the bile duct by *Fasciola* spp., which resulted in hyperplastic cholangitis. Similarly, Matanović *et al*. [28], Hodžić *et al*. [8], and Shrimali *et al*. [6] reported that the high serum GGT level is related to the bile duct epithelium damage induced by adult flukes, cholestasis, and biliary hyperplasia.

Our results showed that the serum urea and creatinine levels were highly elevated in the cattle infected with *F. hepatica*, which may be due to the deposition of granular and pesudolinear immunoglobulin G *F. hepatica* antigen in the mesangial region of the glomeruli that results in membranoproliferative and mesangioproliferative glomerulonephritis in bovine fascioliasis [29].

A significant decrease in the serum IFN-γ level was noted here, which is consistent with the finding reported by Mendes *et al*. [10], who demonstrated suppression of IFN-γ gene expression in the cattle infected with *F. hepatica*. Our results also confirm the findings reported by Zafra *et al*. [30], who showed the absence or very low gene expression of IFN-γ in the hepatic tissue and hepatic lymph node of goats infected with *F*. *hepatica*. Similarly, Molina [31] recorded an absence of serum IFN-γ production in cattle and buffaloes that were experimentally infected with *Fasciola* spp. throughout the 16-week observation period and explained that the T-cell response of these animals involved the apparent Th2 response, with suppression of the Th1 response. This highly polarized immune response may be accompanied by an elevated susceptibility of the host to other pathogens [32]. Our results were also consistent with those reported by Tliba *et al*. [33], who stated that the proportion of IFN-γ-producing cells was significantly lower in rats experimentally infected with *F. hepatica* than in controls on day 14 post-infection.

In the present study, compared with that observed in the control samples, a significant increase in the serum IL-4 and IL-10 levels was noted in the cattle infected with *F. hepatica*. This finding is consistent with that reported by Shi *et al*. [11], who demonstrated a significant increase in the gene expression of IL-10 in cattle naturally infected with *F. hepatica*. Furthermore, Pacheco *et al*. [34] reported an elevated gene expression of IL-10 in sheep experimentally infected with *F. hepatica*. Similarly, Mendes *et al*. [10] found a significant increase in the gene expression of both IL-4 and IL-10 in cattle naturally infected with *F. hepatica*, wherein IL-4 stimulated lymphocyte differentiation into Th2 cells, resulting in the development of fibrosis during the migration and feeding of the parasite. In addition, high expression of IL-4 regulates the effects of IFN-γ, which in turn helps in controlling the number of helminths that reach the hepatic parenchyma and develop into adult worms. The increased levels of both IL-4 and IL-10 were positively correlated, suggesting a synergistic action between these two cytokines in the present study. Consistently, Pleasance *et* *al*. [35] reported significantly increased type 2 cytokine levels (IL-4, IL-5, and IL-13 mRNA expressions) in the hepatic lymph nodes isolated from Indonesian thin-tailed sheep.

Our results demonstrated that compared with the control samples, the cattle infected with *F*. *hepatica* showed a significant increase in the serum TGFβ level. This result partially agrees with that reported by Hacariz *et al*. [36], who observed high IL-10 and TGFβ1 gene expression in sheep experimentally infected with a low amount of liver fluke. IL-10 and TGFβ1 are T-regulatory cytokines that are important in regulating the immune response, and they play a major role in minimizing the pathology and boosting tissue repair during helminth infections. In addition, TGFβ1 is an essential cytokine that promotes collagen production and fibrosis, thereby encapsulating the liver flukes and limiting their evasion into the hepatic parenchyma, which in turn would be beneficial to the host and increase host resistance to *Fasciola* spp.

MDA is one of the end products of lipid peroxidation, which increases with an increasing ROS production [37]. It is used as a biomarker for assessing lipid peroxidation in medical and biological research [38]. The phagocytic response of the liver to *Fasciola* spp. invasion and growth in cattle cause free radical-mediated oxidative stress that initiates and develops with a subsequent increase in MDA level in the hepatic tissue [9]. Our results showed a significant increase in the serum MDA level in the cattle infected with *F*. *hepatica*. These results are consistent with those reported by Bahrami *et al*. [33], who reported that the MDA levels were higher in cattle naturally infected with *Fasciola* spp. than in the non-infected cattle. Similarly, Saleh [3] recorded increased MDA levels in desert sheep naturally infected with *F. hepatica*. Furthermore, Kolodziejczyk *et al*. [39] reported increased MDA levels in the liver of rats experimentally infected with *F. hepatica*.

Oxidation is one of the fundamental factors involved in the non-microbial degradation of meat and meat products. Accordingly, lipid peroxidation has been greatly probed in the all the studied samples, given that the products of lipid peroxidation can actually react with proteins and result in loss of the nutritional value of meat and meat products [40].

Lipid peroxidation is one of the reactions responsible for deterioration of meat and meat products, giving off odors and causing texture modification, rancidity, loss of essential fatty acids, or toxic compound production. In addition, lipid oxidation products have been implicated in several human pathogen-related diseases as well as in cancer, inflammation, atherosclerosis, or aging process [41]. Lipid oxidation affects the levels of fatty acids, particularly polyunsaturated fatty acids, and the oxidation of unsaturated fatty acids in biomembranes can lead to decreased fluidity of the biomembrane and disruption of the normal membrane structure and function [42].

Accordingly, lipid oxidation breaks down the integrity of the biological membrane, which in turn disrupts the water holding capacity [43]. In addition, oxidation is involved in the regulation of MDA, which is a three carbon compound formed by scission of peroxidized polyunsaturated fatty acids of aldehydes, which are formed as end products of lipid peroxidation. Therefore, these compounds react with thiobarbituric acid, which is used to quantify lipid oxidation products by measuring the levels of MDA [44]. MDA is considered a carcinogenic initiator and can hence affect the safety of food [42].

The imbalance between pro-oxidants and endogenous antioxidant prompt the excessive production of free radicals, such as ROS and reactive nitrogen species. It is known that an overflow of free radicals elicits oxidative stress, resulting in harmful effects on cellular biomacromolecules such as DNA, protein, and lipids [45].

Free radicals accumulated in response to oxidative stress impede the cell membrane and mitochondrial integrity through lipid peroxidation, which substantially increases the risk of oxidative reactions during the postmortem aging of meat products [46]. Moreover, the meat becomes susceptible to oxidative process due to the high levels of unsaturated fatty acids and various indicators such as transition metals, pigments, and certain oxidoreductase enzymes. Thus, oxidation has been described as a major cause affecting muscle protein function and the nutritional, sensory, and shelf life quality of animal products [47].

Lipid peroxidation is a free radical-mediated chain of reaction. ROS, such as hydroxyl radical (^–^OH), hydrogen peroxide (H_2_O^–^), superoxide anion (O_2_^–^), and hydroperoxyl radical (H_2_O_2_^–^), are the most prominent initiators of these chain reactions. Protein oxidation involves direct reaction with an ROS or an oblique reaction with secondary products of lipid peroxidation, leading to the covalent modification of a protein [48]. The oxidative modification of muscle protein causes considerable physical and chemical changes in their properties, such as conformation, bioavailability, solubility, aggregation, and capability to undergo proteolysis. All these relationships can affect the basic quality traits, nutritional values, and functionalities of meat [47].

*Fasciol*a spp., which is considered as one of the extrinsic stressors, may affect the normal physiological equilibrium and thus the stress response. Thereafter, the animal’s physiological and metabolic functions, which dominate the postmortem biochemical changes, are affected. As a result, the intensity of the stressor and the susceptibility of the animal to the stressors are of major significance in animal welfare and meat quality development [49]. In the present study, NO level was significantly increased in the cattle infected with *Fasciola* spp. compared with that in the control samples. These results are consistent with those reported by Bottari *et al*. [50], who reported high NOx levels in the liver of rats experimentally infected with *F. hepatica*, which causes cytotoxicity to cells due to the ability of NO to generate peroxynitrite, with subsequent initiation of various oxidative reactions, including modification of nucleic acids, lipids, and proteins, which causes tissue injury.

Recently, NO-induced protein nitrosylation and oxidation have also been found to play a significant role in mediating the quality of fresh meat. Preslaughter regulation of NOs induces nitrolysation, which can affect the postmortem meat tenderness [51]. In addition, Zhang *et al*. [47] observed that the pectoralis muscle of broilers incubated with an NO inducer showed changes in the oxidative status and postmortem meat quality.

Our results demonstrated a significant decrease in GSH levels in the infected cattle compared with that in the control samples, which are consistent with the findings reported by Kolodziejczyk *et al*. [39], who demonstrated increased MDA levels in the liver of rats experimentally infected with *F. hepatica* due to increased oxidation of GSH into GSH disulfide, which is catalyzed by free radicals. Furthermore, the decrease in GSH levels found in the present study may compromise cellular antioxidant defenses and lead to ROS accumulation. These results are in agreement with those reported by Saleh [3], who demonstrated decreased plasma GSH levels in desert sheep naturally infected with *F. hepatica* as a result of decreased reserves of the antioxidants, which exaggerate the generation of free radicals, with subsequent enhancement of lipid peroxidation. The observed decrease in GSH levels will decline the meat quality of slaughtered animals. Given that GSH acts as an antioxidant during the postmortem oxidation of proteins (e.g., oxymyoglobin to metmyoglobin), this action plays an utmost role in preserving the shelf life and quality of meat products, in addition to improving color stability [52].

The Egyptian veterinary authorities mention that the liver should be completely condemned while investigating fascioliasis in the liver during the postmortem examination at abattoirs; however, in case of partial infestation, parts of the lobes can be removed. Thereafter, the remaining parts of the liver and the entire carcass are released for human consumption after approval by applying the rapid phase test and boiling test [53].

The direct economic losses because of the liver condemnation as a result of the presence of the pathological lesions due to bovine fascioliasis have been reported to be 1 million dollars. The difference between the direct economic losses [54] due to bovine tuberculosis and bovine fascioliasis at the same abattoirs and at the same period may possibly clarify the results reported by Ibironke and Fasina [54] stating that more liver was condemned due to fascioliasis than due to other diseases.Despite the limitation of using meat inspection data, not being able to assign the effect of fascioliasis on keeping quality of the liver as one of the most important offals rich with different nutrients. The analysis presented will provide useful information for further testing to evaluate the effect of fascioliasis on the offals and meat production.

## Conclusion

The present study reported a prevalence rate of fascioliasis caused by *F. hepatica* at 16.81% in 226 cattle at the El-Kharga abattoir, as revealed by postmortem examination. The analysis of the biochemical and immunological parameters indicated the damage caused to liver tissue and the bile duct due to *F*. *hepatica* infection. *F. hepatica* is one of the extrinsic oxidative stress factors that affect slaughtered animals, causing biochemical and metabolic changes in the early postmortem period. These changes have serious effects on both animal welfare and quality aspects.

## Authors’ Contributions

NN and RSZ collected the samples, carried out the examination and the experiments. In addition, they drafted the manuscript, conducted data analysis and revised the manuscript. Both authors read and approved the final manuscript.
